# P-1916. The impact of SARS-CoV-2 VOCs on clinical outcomes: an Umbrella Review

**DOI:** 10.1093/ofid/ofae631.2076

**Published:** 2025-01-29

**Authors:** Federico Fama, Rebecca Fattore, Paolo Raimondo, Fabio Brivio, Darcy Holmes, Toussaint Muheberimana, Tarek Nayfeh, Andrea Gori, Matteo Passerini, Marta Colaneri

**Affiliations:** Infectious Diseases and Immunopathology, Department of Clinical Sciences, Università di Milano, Luigi Sacco Hospital, Milan, Italy, Milan, Lombardia, Italy; Infectious Diseases and Immunopathology, Department of Clinical Sciences, Università di Milano, Luigi Sacco Hospital, Milan, Italy, Milan, Lombardia, Italy; Infectious Diseases and Immunopathology, Department of Clinical Sciences, Università di Milano, Luigi Sacco Hospital, Milan, Italy, Milan, Lombardia, Italy; Infectious Diseases and Immunopathology, Department of Clinical Sciences, Università di Milano, Luigi Sacco Hospital, Milan, Italy, Milan, Lombardia, Italy; Department of Pathophysiology and Transplantation, University of Milano, Milan, Italy, Milan, Lombardia, Italy; Centre for Multidisciplinary Research in Health Science (MACH), University of Milan - Milan, Italy, Milan, Lombardia, Italy; Division of Public Health, Infectious Diseases, and Occupational Medicine, Mayo Clinic, Rochester, USA, Rochester, Minnesota; Infectious Diseases and Immunopathology, Department of Clinical Sciences, Università di Milano, Luigi Sacco Hospital, Milan, Italy, Milan, Lombardia, Italy; Department of Pathophysiology and Transplantation, University of Milano, Milan, Italy, Milan, Lombardia, Italy; 1. Department of Infectious Diseases, Luigi Sacco University Hospital – Milan, Italy, Milan, Lombardia, Italy

## Abstract

**Background:**

Studies aiming to synthetize data from literature are helpful to valid the robustness of associations, to assess the quality of the data, and, eventually, to propose recommendations. This is particularly evident for the huge amount of data reported for SARS-CoV-2.

The aim of this study was to perform an umbrella review to identify the strength and validity of associations between variants of concern (VOCs) and pre-specified clinical outcomes in COVID-19 patients.

Table 1

**Figure:**
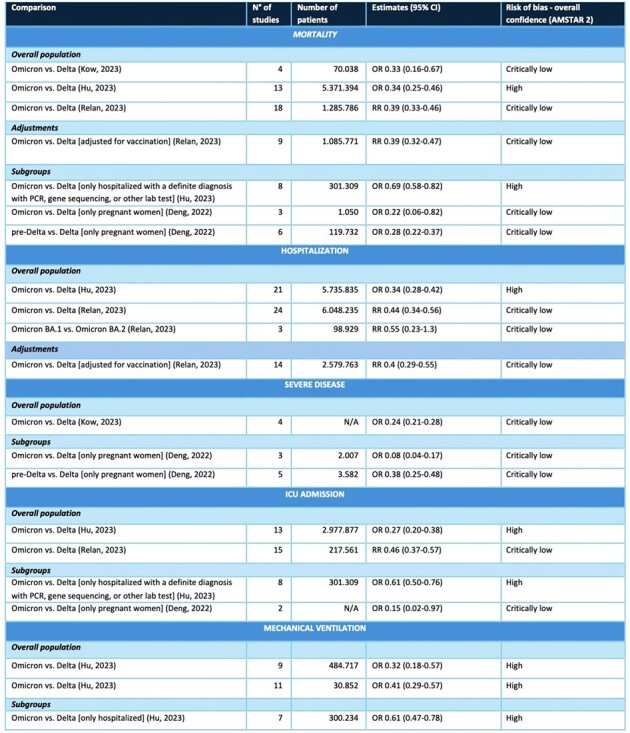

Effect size measures reported in the studies included in the review, divided by outcome.

Acronyms: CI, confidence interval; OR: odds ratio; RR, risk ratio; cCFR, confirmed case-fatality risk; HFR, hospitalization-fatality risk; N/A, not available

**Methods:**

An umbrella review according to the principles to Preferred Reporting Items for Systematic Reviews and Meta-Analyses (PRISMA) was performed searching multiple databases in January 2024. Peer reviewed systematic reviews considering ≥ 2 VOCs with a clinical outcome among death, hospitalization, severe disease, admission to intensive care unit (ICU), and mechanical ventilation were included. Data on study population and measures of association between clinical outcome and VOCs were considered. The quality of the studies was assessed through the AMSTAR-2 tool. The effect size measures with the confidence interval for each association between VOCs and clinical outcomes were reported. Subgroup analyses were performed, if possible. A citation matrix was used to assess the overlap between the systematic reviews included.

Table 2

**Figure:**
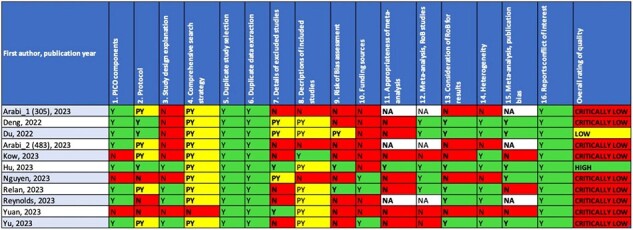

Risk of bias assessment of the included systematic reviews using the AMSTAR-2 score. In bold the key domains.

**Results:**

A total of 11 studies were included in our review. 24 total comparison were considered, most regarding Omicron versus Delta (19/24), where Omicron was always associated with better clinical outcomes than Delta. The overall quality of the studies was low. None of the reviewed studies incorporated consideration of risk factors other than VOCs in evaluating clinical outcomes.

The overlap between the included reviews is low, only 10% of them having a significant overlap ( > 15%).

**Conclusion:**

Our umbrella review shows the lower hazard on human health of the Omicron compared to Delta variant. However, the quality of the reviews included were generally low and there is a lack of correction with other risk factors such as comorbidities and vaccination status.

While our review provides valuable insights into the association between VOCs and clinical outcomes of SARS-CoV-2 infection, further research incorporating risk factor analysis and ensuring methodological rigor is warranted.

**Disclosures:**

All Authors: No reported disclosures

